# A chemiresistive sensor array based on polyaniline nanocomposites and machine learning classification

**DOI:** 10.3762/bjnano.13.34

**Published:** 2022-04-27

**Authors:** Jiri Kroutil, Alexandr Laposa, Ali Ahmad, Jan Voves, Vojtech Povolny, Ladislav Klimsa, Marina Davydova, Miroslav Husak

**Affiliations:** 1 Department of Microelectronics, Czech Technical University in Prague, Technicka 2,166 27 Prague, Czech Republic; 2 FZU - Institute of Physics, of the Czech Academy of Sciences, Na Slovance 1999/2, 182 21 Prague, Czech Republic

**Keywords:** feature extraction, gas sensor, pattern recognition, sensor array

## Abstract

The selective detection of ammonia (NH_3_), nitrogen dioxide (NO_2_), carbon oxides (CO_2_ and CO), acetone ((CH_3_)_2_CO), and toluene (C_6_H_5_CH_3_) is investigated by means of a gas sensor array based on polyaniline nanocomposites. The array composed by seven different conductive sensors with composite sensing layers are measured and analyzed using machine learning. Statistical tools, such as principal component analysis and linear discriminant analysis, are used as dimensionality reduction methods. Five different classification methods, namely *k*-nearest neighbors algorithm, support vector machine, random forest, decision tree classifier, and Gaussian process classification (GPC) are compared to evaluate the accuracy of target gas determination. We found the Gaussian process classification model trained on features extracted from the data by principal component analysis to be a highly accurate method reach to 99% of the classification of six different gases.

## Introduction

The control and monitoring of toxic gaseous substances, such as ammonia, nitrogen oxides, and various volatile organic compounds, is crucial in automotive, defense, aviation, chemical, medicine, and food industries [1–2]. Research on chemical sensors is currently focused on the fabrication of multisensor arrays for enhanced detection and identification of various chemical compounds. In most cases, the cross-sensitivity toward different chemical analytes is unavoidable, regardless of their oxidizing or reducing nature. Many authors have suggested a number of ways to overcome the drawbacks of cross-sensitivity/selectivity and reliability of the sensor arrays [3–5]. One powerful tool to address the abovementioned drawbacks is the implementation of a multisensor array combined with appropriate pattern recognition and classification tools [6]. Recently, classification in gas sensing applications has been carried out by principal component analysis to identify the difference between VOCs, supported by a vector machine to distinguish between acetone, nitrogen dioxide, and ammonia, and by a neutral network model to distinguish between ammonia and formaldehyde gas [7–9].

In our previous work [10], we demonstrated a combination of organic (polyaniline, PANI) and inorganic (carbon nanotubes (CNT), SnO_2_, TiO_2_) materials in a gas sensors based on nanocomposite layers with good sensitivity, temperature stability, reversibility, which was operating at room temperature. Herein, we extended our study by applying other nanocomposite sensing layers, namely PANI/ZnO, PANI/WO_3_ (nanopowder), PANI/WO_3_ (nanotubes), PANI/In_2_O_3_, PANI/C_60_ (fullerene), PANI/nanocrystalline diamond (NCD), and PANI/BaTiO_3_, deposited on a flexible sensor array platform with a new design. Seven different nanocomposite sensing layers deposited on the array were exposed to six different gases (ammonia, carbon dioxide, nitrogen dioxide, carbon monoxide, acetone, and toluene). Moreover, the obtained data were used for machine learning classification.

Many pattern recognition models based on intuitive, linear and nonlinear supervised techniques have been explored in E-nose data [11–12]. A considerable number of studies have been implemented in recent years using different statistical analysis algorithms, like principal component analysis (PCA), linear discriminant analysis (LDA), *k*-nearest neighbors algorithm (KNN), support vector machine (SVM), decision tree classifier (DT), random forest (RF), and Gaussian process classification (GPC) in order to enhance the discrimination of gases and get better selectivity [13]. In this work we suggest a new method in our classification system by combining the abovementioned methods and using the output of the two most powerful techniques in dimensionality reduction and increasing interpretability. We apply PCA and LDA as input data for five machine learning algorithms with a 10-fold cross-validation method.

The preprocessing stage was implemented by applying PCA and LDA on the extracted dataset [14–15]. Five different kinds of flexible pattern recognition algorithms have been used for the classification of gas sensor data using a 10-fold cross-validation to reach the highest classification rate.

## Results and Discussion

The sensors layers were investigated by scanning electron microcopy (SEM), Raman spectroscopy, current–voltage and temperature analysis, and gas sensing analysis. Further, statistical classification analysis was implemented for the evaluation of target gases.

### Scanning electron microscopy and Raman spectroscopy

**Figure 1:**
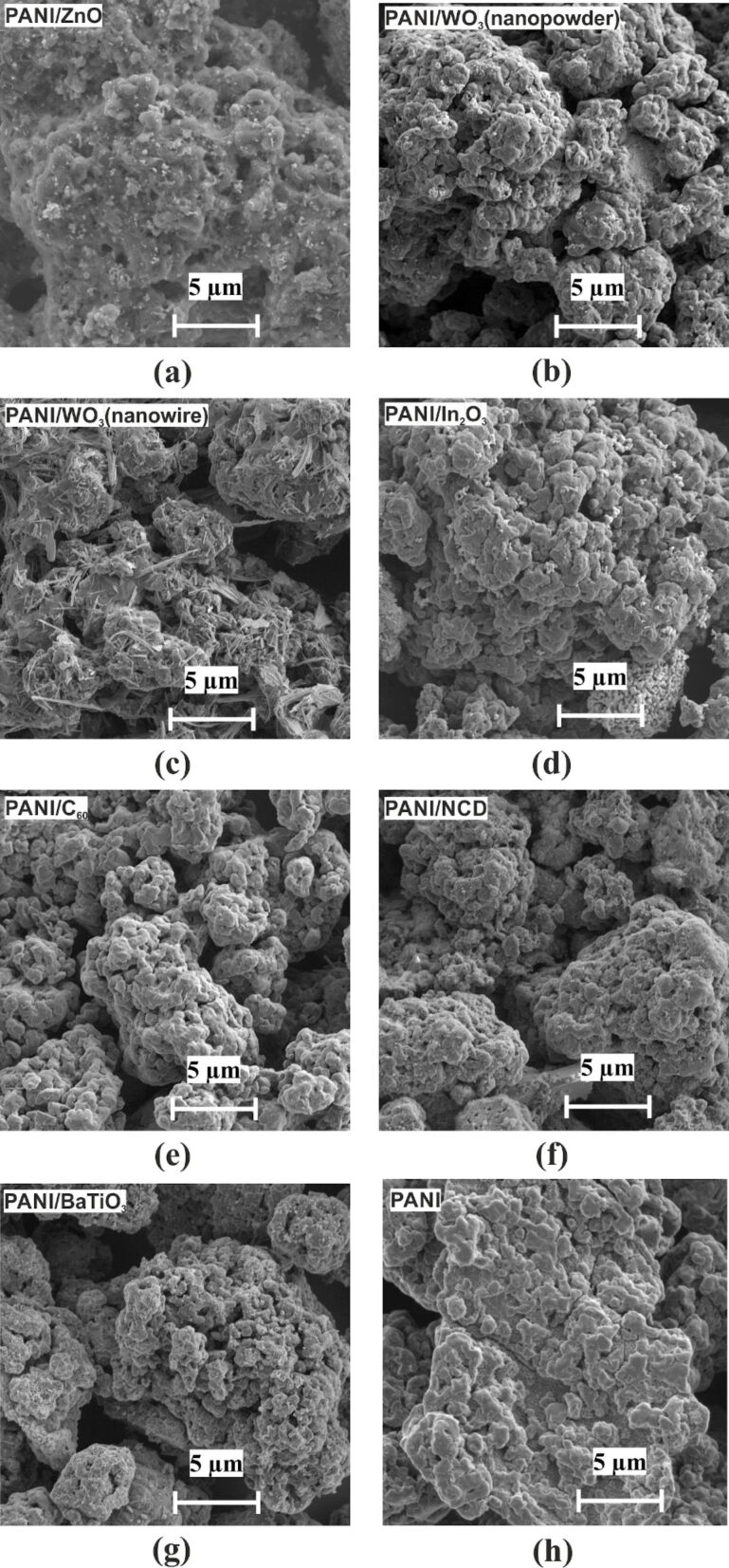
SEM micrographs of deposited layers on an interdigital transducer structure: (a) PANI/ZnO, (b) PANI/WO_3_ (nanopowder), (c) PANI/WO_3_ (nanowire), (d) PANI/In_2_O_3_, (e) PANI/C_60_, (f) PANI/NCD, (g) PANI/BaTiO_3_, and (h) pristine PANI.

The surface morphology and uniformity of additives in PANI of the deposited active layers were examined by scanning electron microscopy (TESCAN FERA3 GM), as shown in Figure 1a–h. All nanocomposite active layers, that is, PANI/ZnO, PANI/WO_3_ (nanopowder), PANI/WO_3_ (nanowires), PANI/In_2_O_3_, PANI/C_60_, PANI/NCD, and PANI/BaTiO_3_ have similar morphological features with a uniform distribution of additives in polyaniline. We can observe small ZnO flakes and WO_3_ nanowires homogeneously distributed in the layers.

**Figure 2:**
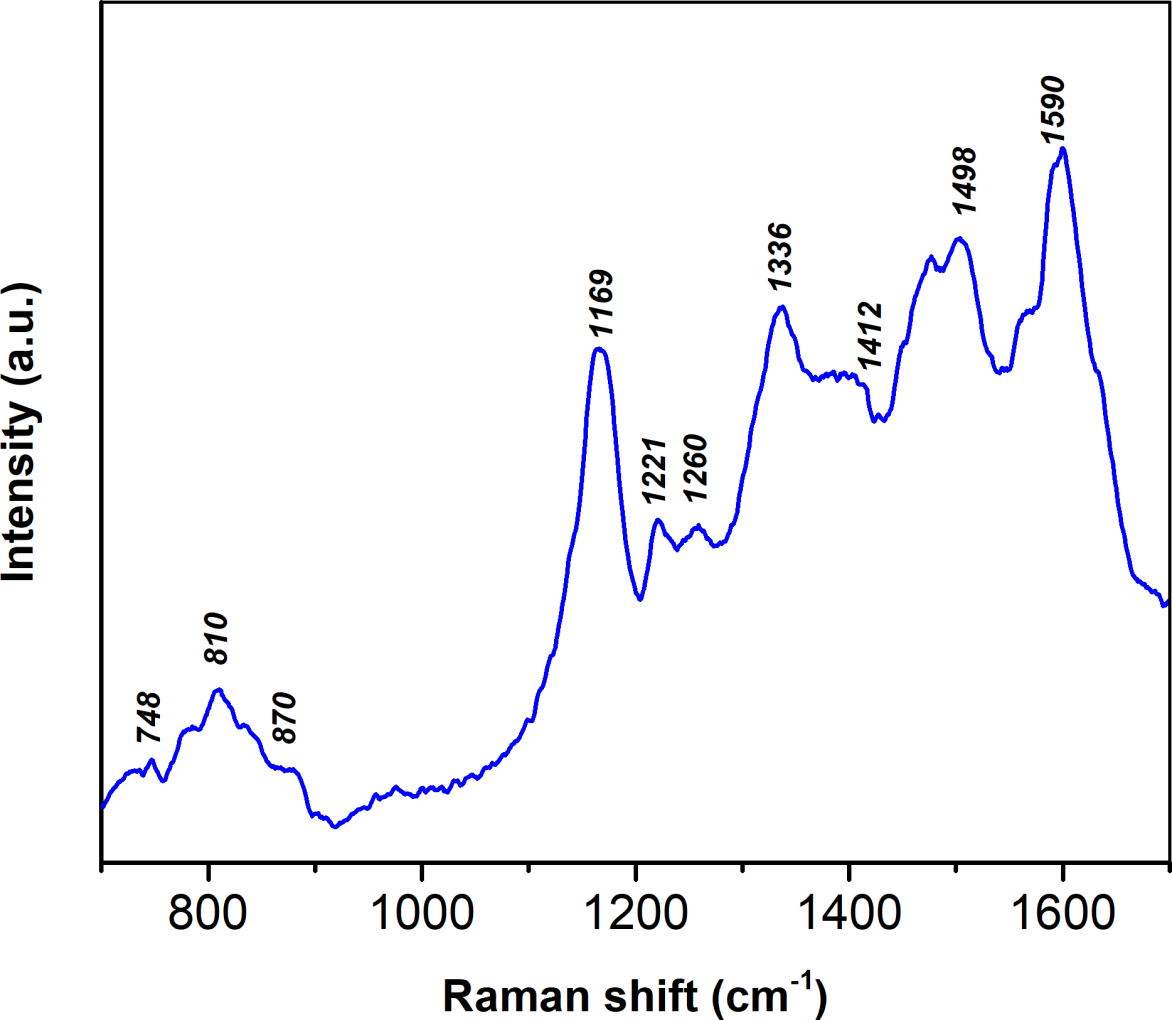
The Raman spectra of the PANI.

Pristine PANI was examined by SEM (Figure 1h) and Raman spectroscopy (Raman spectrometer Renishaw inVia Qontor) at room temperature with 633 nm excitation wavelength (Figure 2). The spectrum of pristine PANI is typical of the emeraldine salt, showing the following main bands: (1) 748 cm^−1^ (Q ring bending, C–C ring deformation); (2) 810 and 870 cm^−1^ (out-of-plane C–H vibrations in the aromatic rings); (3) 1169 cm^−1^ (C–H bending of the quinoid rings); (4) 1221 and 1260 cm^−1^ (C–N in benzene diamine units); (5) 1336 cm^−1^ (C–N^+^, characteristic band of the polaron radical cation); (6) 1412 cm^−1^ (phenazine structures); (7) 1498 cm^−1^ (C=N of the quinoid nonprotonated diimine units); (8) 1590 cm^−1^ (C=C stretching vibration of the quinonoid ring) [16–17].

### Current–voltage and temperature analysis

**Figure 3:**
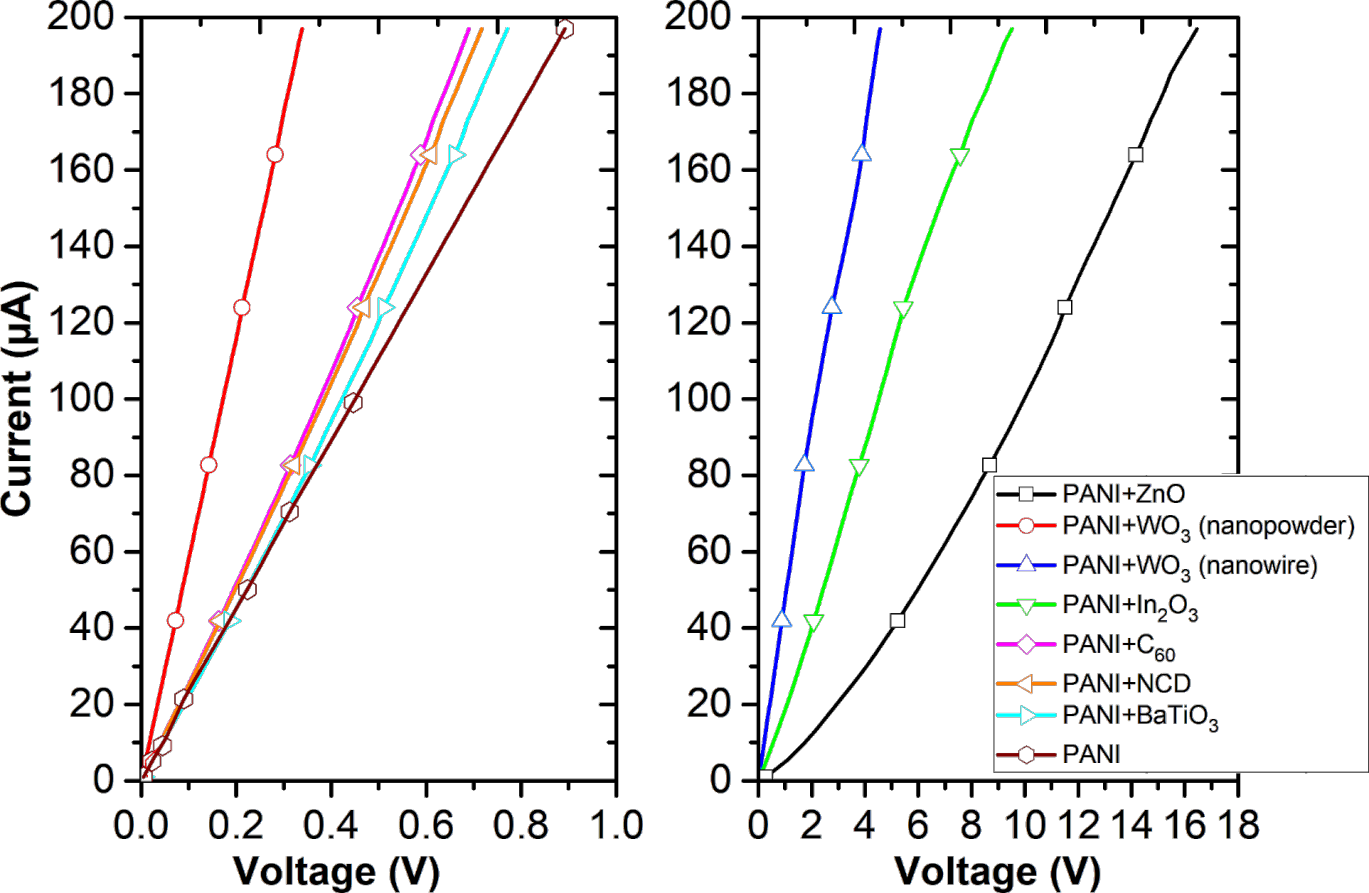
Current–voltage characteristics of active layers.

Figure 3 shows current–voltage characteristics of active layers. These characteristics were examined for currents up to 200 mA and exhibit an almost linear character.

**Figure 4:**
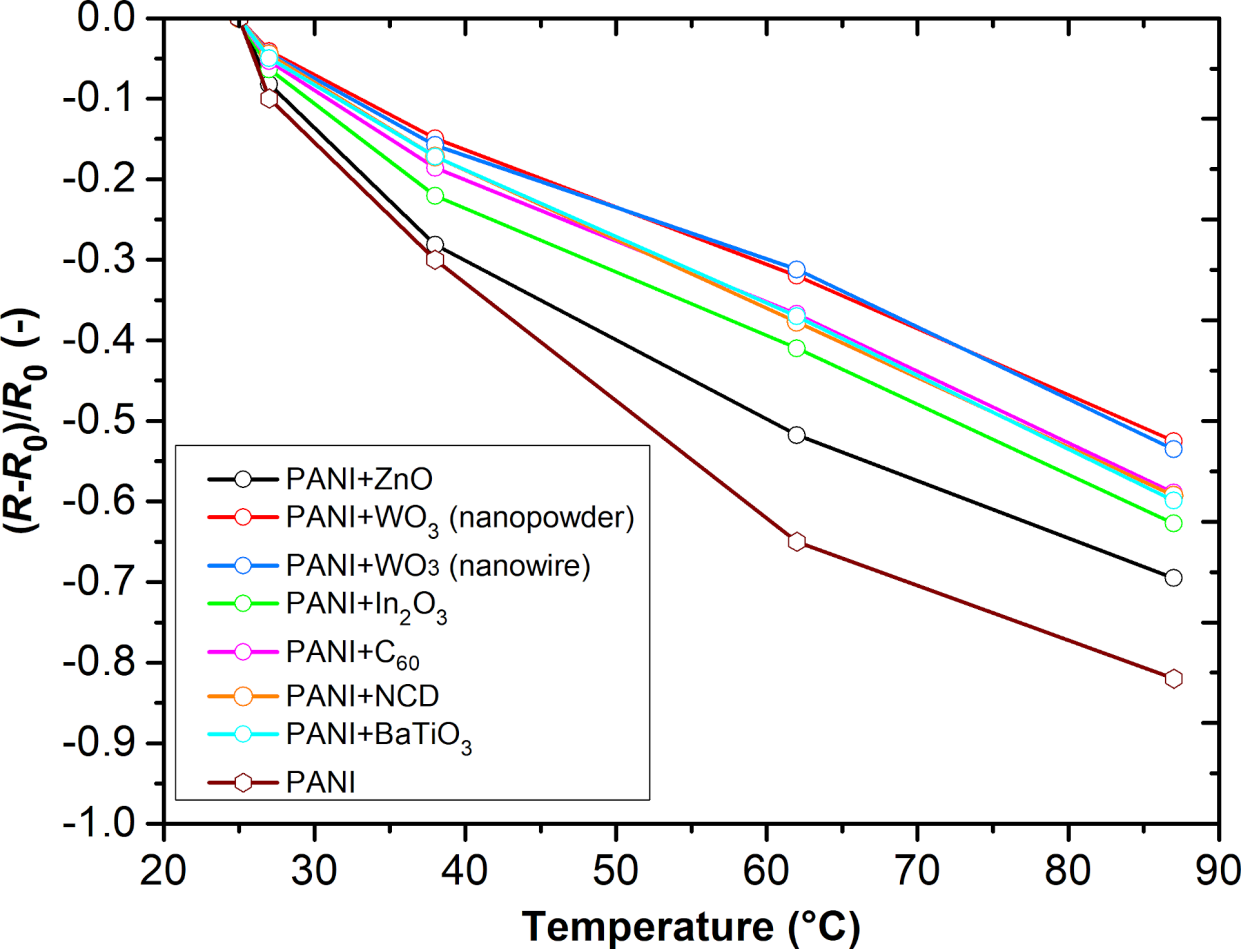
Temperature dependence characteristics of active layers.

Figure 4 shows the temperature dependence of the relative resistance of PANI/nanocomposite layers for different temperatures. All layers exhibit a decrease in resistance with increasing temperature and composite layers exhibit lower temperature dependences in comparison to a pristine PANI layer. The resistance *R*_0_ and the average temperature coefficient of resistance (TCRs) values of the prepared sensing layers are given in Table 1. All active layers have a negative temperature coefficient. The resistivity of these nanocomposites depends on the p–n depletion layer width on the interface between the n-type nanoparticles and the surrounding p-type PANi molecules.

**Table 1:** Average TCRs of prepared sensitive layers in range from 20 to 80 °C.

Layer	*R*_0_ (Ω)	TCR (K^−1^)

PANI+ZnO	85123	−0.012
PANI+WO_3_ (nanopowder)	1764	−0.009
PANI+WO_3_ (nanowires)	25906	−0.009
PANI+In_2_O_3_	50750	−0.011
PANI+C_60_	3305	−0.01
PANI+NCD	3549	−0.01
PANI+BaTiO_3_	4338	−0.0096
PANI	4240	−0.012

### Gas sensing analysis

**Figure 5:**
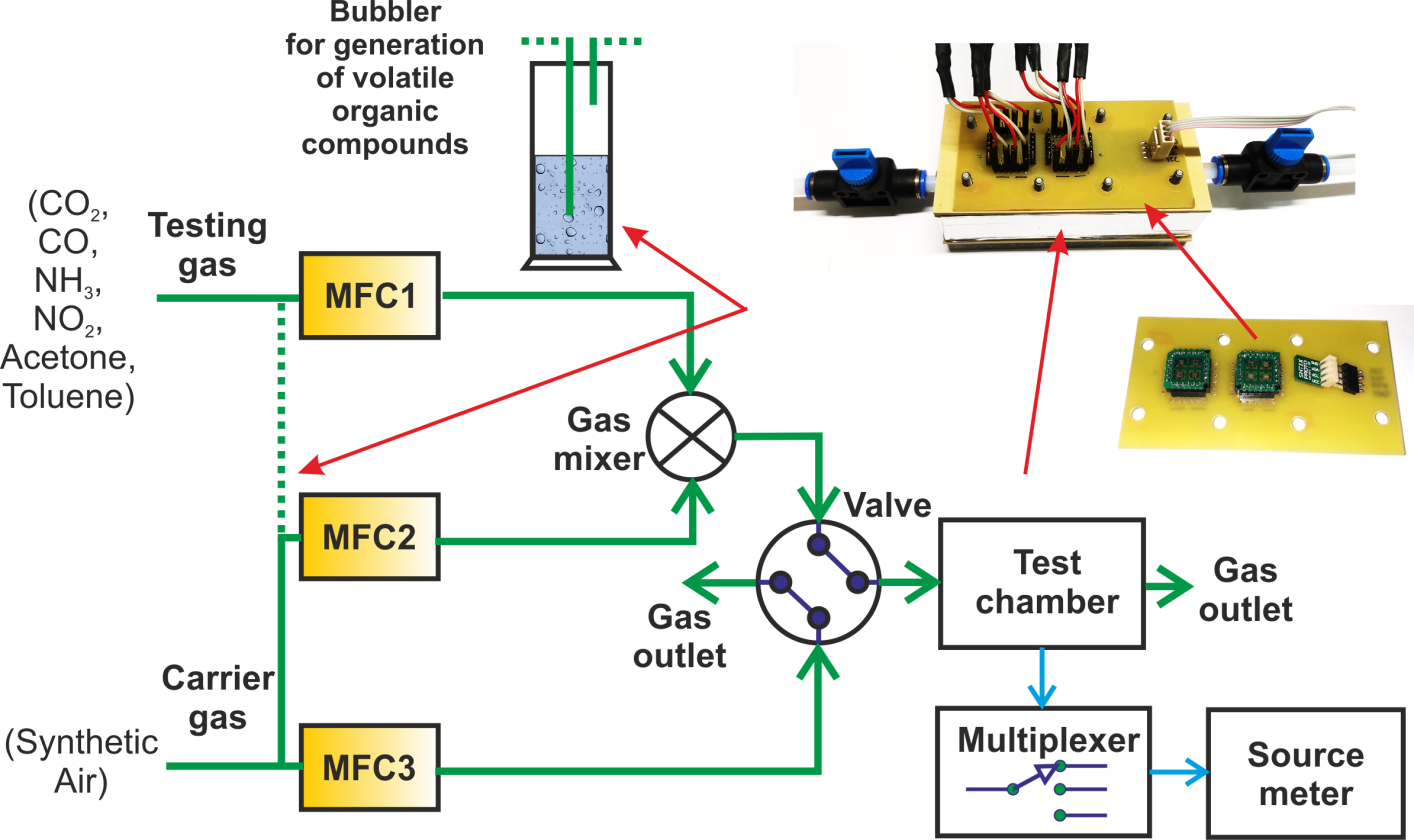
Schematic diagram of the gas sensing characterizations apparatus.

The gas sensing characterizations of sensitive layers were performed using a custom-built apparatus (Figure 5). The characterization system consists of mass flow controllers (Bronkhorst High-Tech) for setting the required gas concentration, a source-meter (Keithley, Model 2400) for resistance measurement of active layers, an air-tight chamber with electrical feedthroughs for the sensor array, a relay multiplexor for switching four sensor elements, and a Labview-based data acquisition system.

The sensing layers were tested towards carbon dioxide (250 ppm CO_2_), carbon monoxide (25 ppm), ammonia (25 ppm), nitrogen dioxide (25 ppm), acetone (6%), toluene (500 ppm) and humid air (RH) in synthetic air (SA) at room temperature. The response of the sensing layers was calculated by the relative resistance change:


[1] $$\frac{\Delta R}{R_{0}}=\frac{R_{0}-R_{\text{g}}}{R_{0}},$$

where *R*_0_ is the resistance at room temperature in synthetic air and *R*_g_ is the resistance of the sensor in the presence of the specific gas.

**Figure 6:**
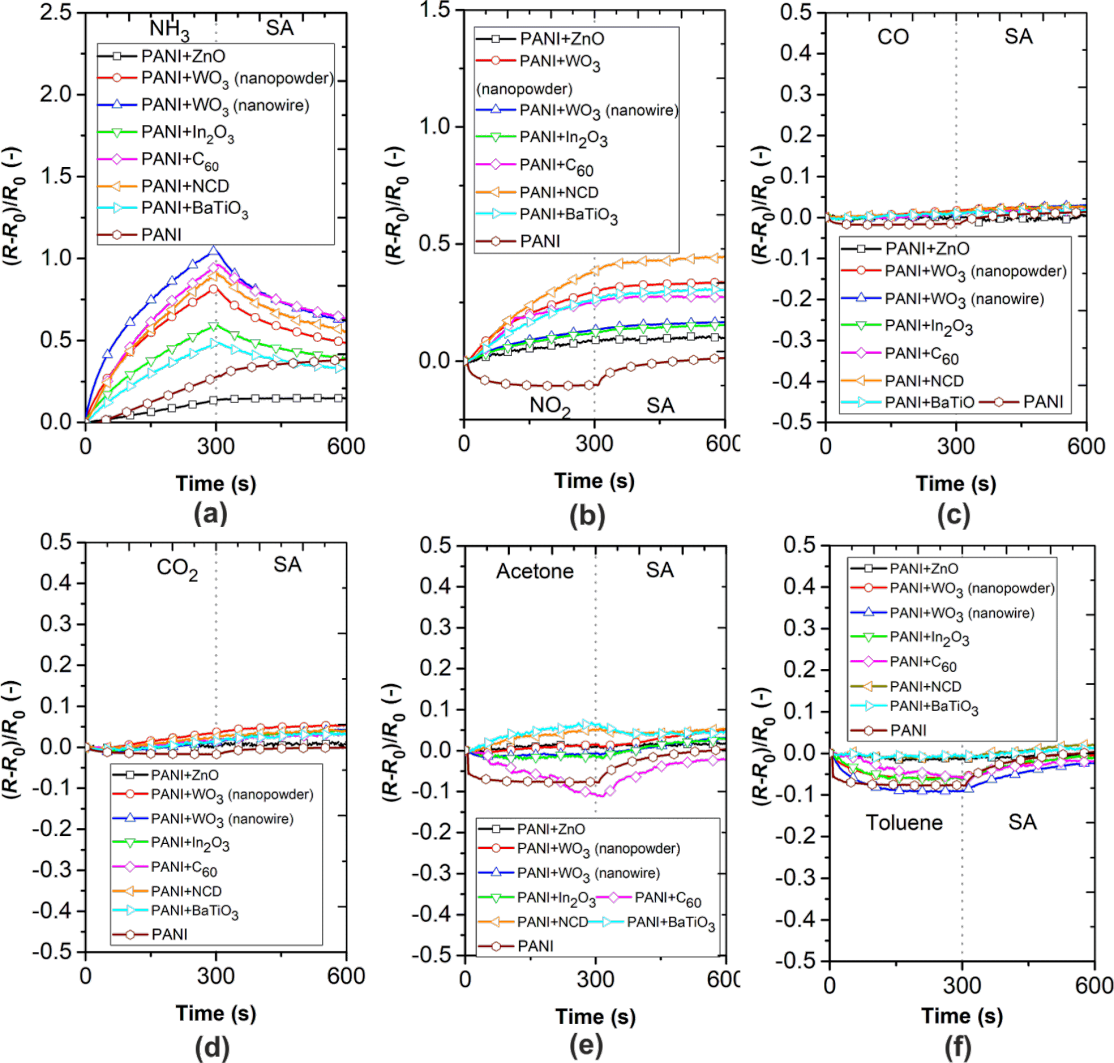
Gas characterization of active layers towards (a) 25 ppm of NH_3_, (b) 25 ppm of NO_2_, (c) 25 ppm of CO, (d) 250 ppm of CO_2_, (e) 6000 ppm of acetone, and (f) 500 ppm of toluene at room temperature.

The dynamic responses of sensing layers for gases are displayed in Figure 6. The experiment consisted of 5 min of sensor exposure to a certain gas concentration and 5 min of purging at a flow rate of 100 mL·s^−1^. It is evident that all active layers have the highest sensitivity and clear response to NH_3_. All active composite layers except PANI+ZnO exhibit a higher sensitivity toward NH_3_ in comparison to a pristine PANI layer. PANI+ZnO composite shows the lowest sensitivity to all gases. Moreover, the resistance decreases when the polyaniline composite sensing layers are exposed to toluene. In addition, the sensor responses of all sensing layers to NO_2_ gas are nearly three times lower than those to NH_3_.

**Figure 7:**
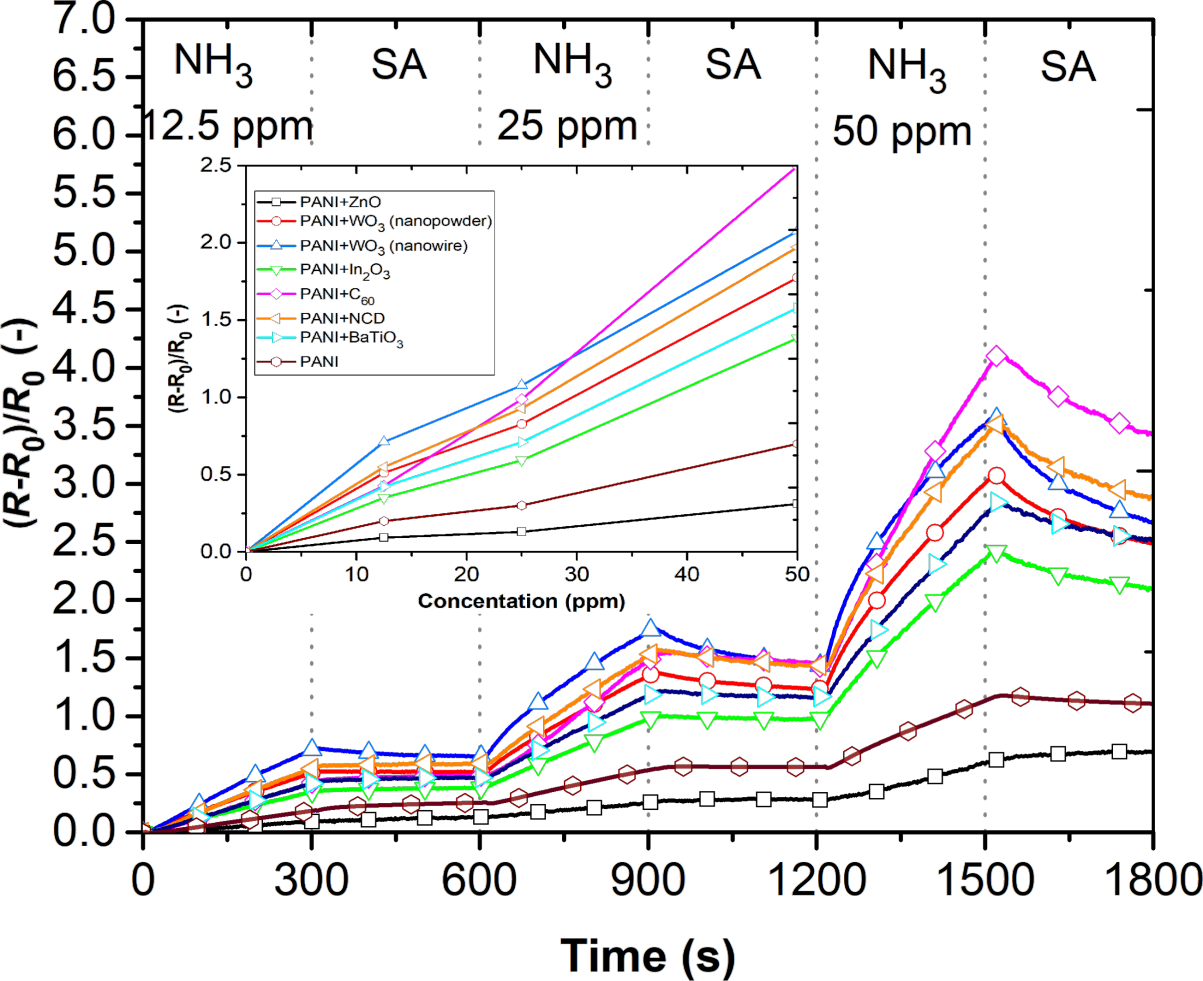
The responses of sensing layers for different concentrations of NH_3_.

Due to the high sensitivity to NH_3_ gas, the sensing layers were also tested towards different concentrations of NH_3_ at room temperature (Figure 7). It is evident that all sensing layers demonstrate an enhancement in sensitivity to the highest gas concentration (50 ppm) as well as incomplete reversibility.

**Figure 8:**
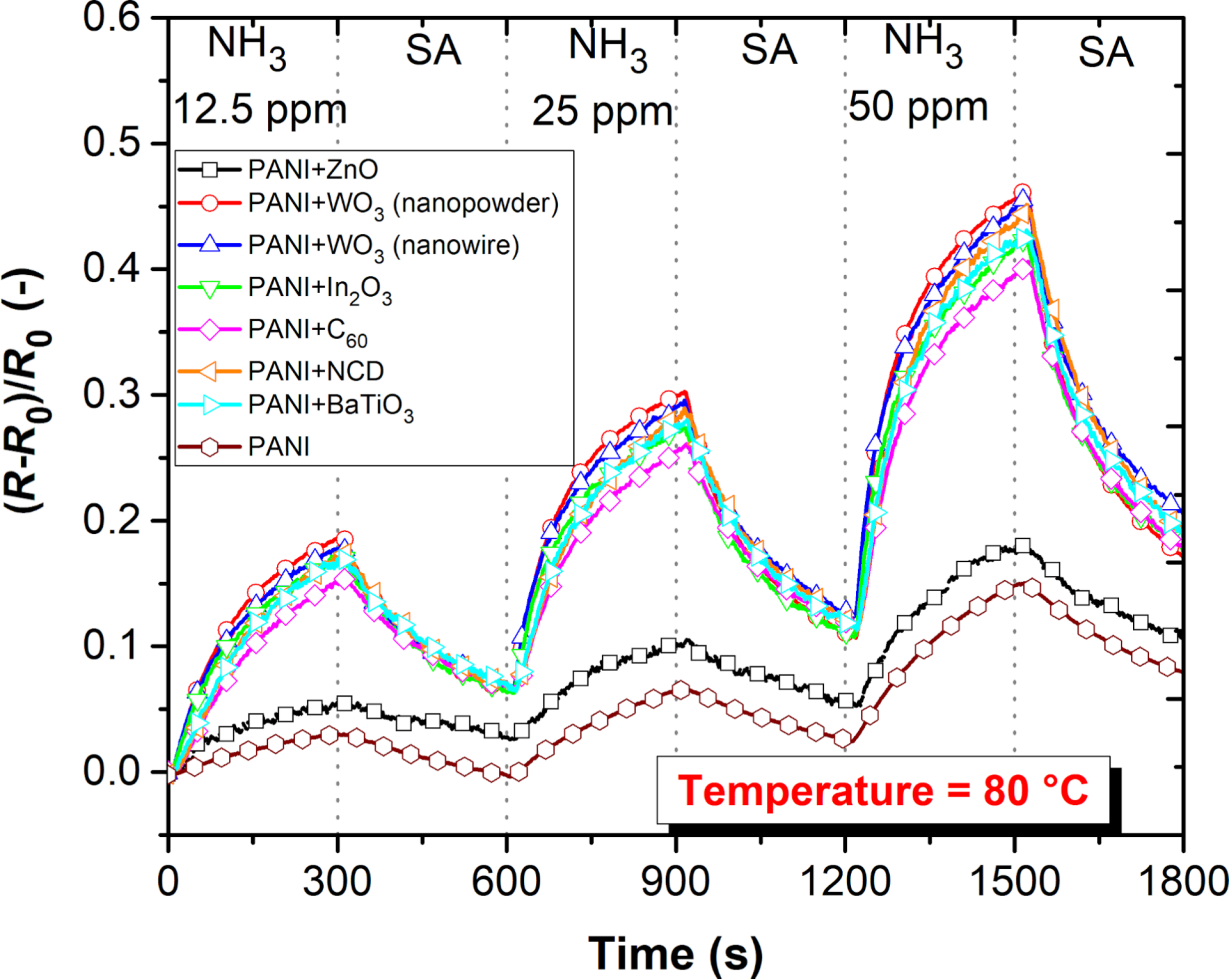
Gas characterization of active layers to different concentrations of NH_3_ at 80 °C.

When the operating temperature increased up to 80 °C, the sensing layers showed an at least three times lower response to ammonia in contrast to the room temperature measurements (Figure 7), but with almost complete reversibility (Figure 8).

**Figure 9:**
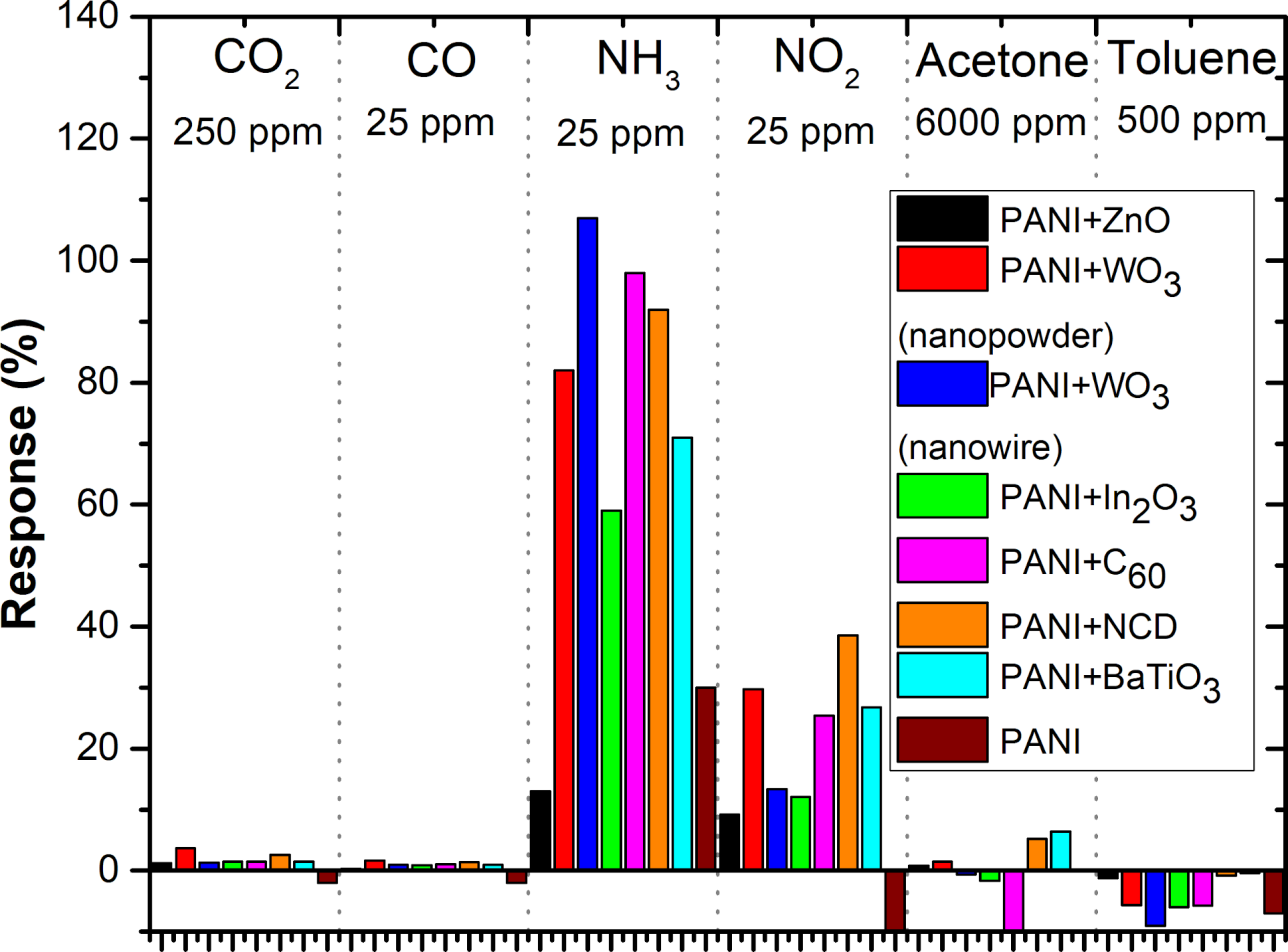
The summary of the gas sensor responses for all active layers.

The gas sensing mechanism of nanocomposite layers based on PANI was discussed in [10]. Polyaniline is known as one of the most famous p-type conductive polymers. During the exposure to a reducing gas (NH_3_), the emeraldine salt form of polyaniline is converted to the emeraldine base form leading to an increase in resistance due to the decrease of hole density within the p-type film [18]. In the case of the hybrid structures, a p–n heterojunction is formed between polyaniline and n-type nanostructures such as ZnO, WO_3_, In_2_O_3_, or fullerene [19]. The protons from polyaniline are transferred to the NH_3_ molecules. This results in a widening of the depletion layers on p–n junctions and, thus, the resistance increases [18]. All these effects are reversible when the reducing gas is replaced by air. Beside these effects, nanostructures added into polyaniline increase the initial resistance due to larger disorder and deformation of the polyaniline conjugation chains. The summary of the gas sensor responses for all active layers is shown in Figure 9. All layers show an increasing resistivity as a clear response to CO, CO_2_, NH_3_, and NO_2_.

**Figure 10:**
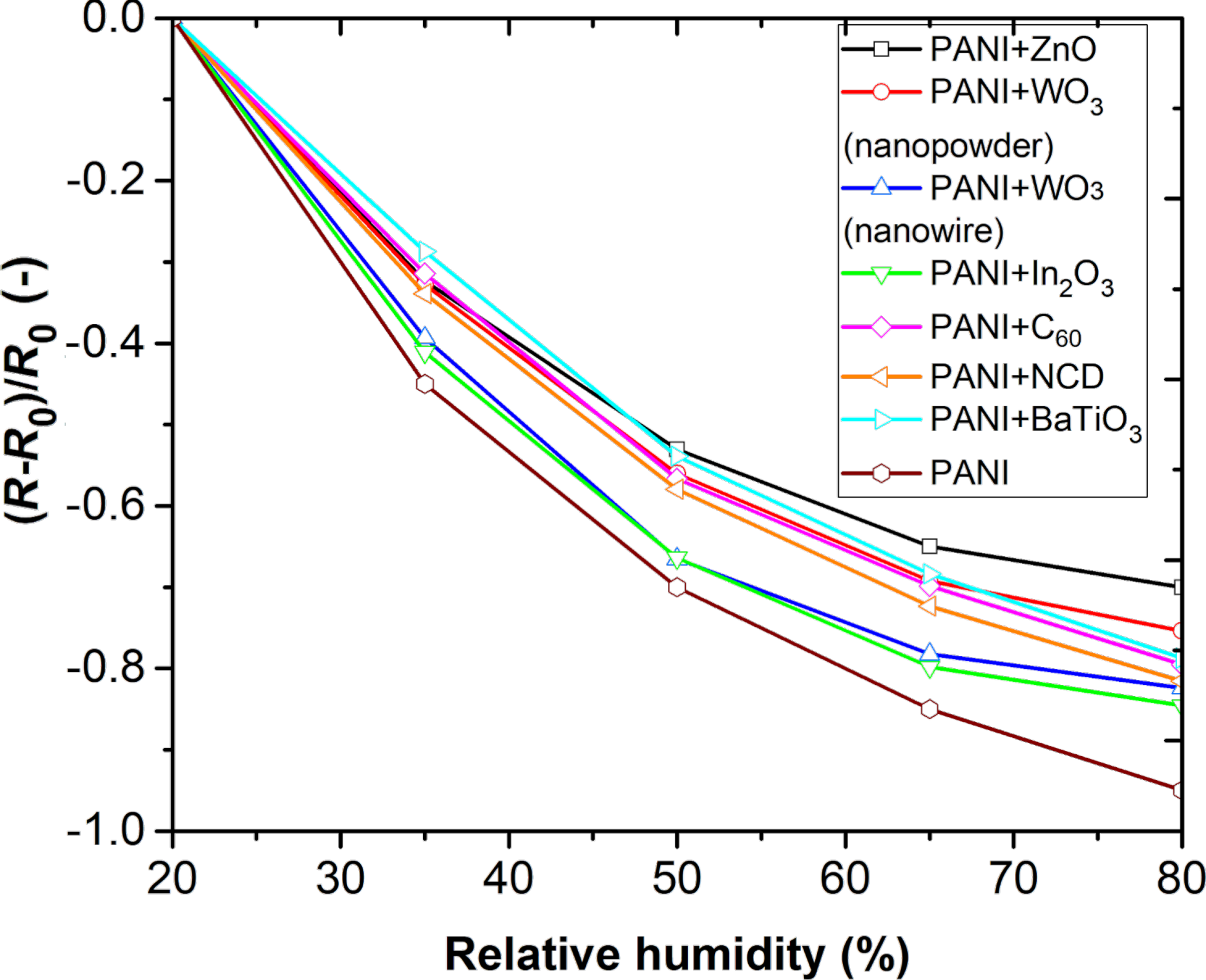
RH dependences of the sensing layers.

Additionally, to exclude the influence of humidity on the sensor signal, the sensor arrays were exposed to various relative humidity levels ranging from 20% to 80% (Figure 10). The obtained results show a decreasing sensor response with increasing RH% value. This change in response toward relative humidity is due to the adsorption of water molecules and an increase of charge concentration due to PANI doping and the formation of charge transfer complexes [20]. The decrease of electrical resistance is caused by the greater mobility of the dopant ions, related to the development of PANI chains. Furthermore, the swelling effect contributes to the change in resistivity [21].

### Statistical classification analysis

**Figure 11:**
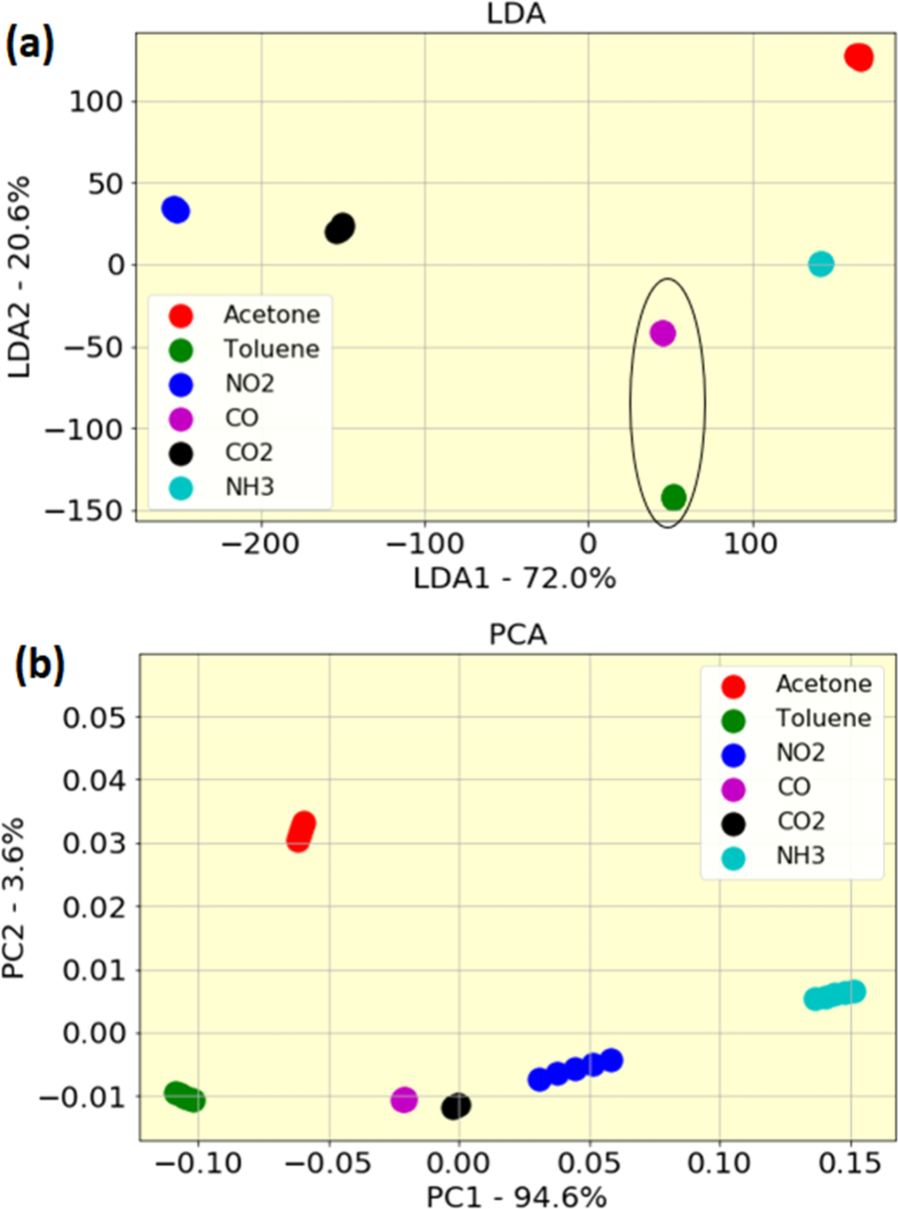
Two-dimensional (a) LDA and (b) PCA projections of extracted features.

A data preprocessing stage was applied on the gas sensor responses to improve the overall pattern analysis performance. It was implemented by applying PCA and LDA on the extracted dataset of exposing six different gases to sensor arrays with seven different PANI/nanocomposite sensing layers. Figure 11 presents two-dimensional LDA and PCA projections of the extracted features. Regarding the LDA results, LDA1 describes approximately 72% and LDA2 describes 20.6% of data variation. By using the LDA method, a good classification with clearly separated clusters is obtained for NO_2_, CO_2_, acetone, and NH_3_, but an overlap (overlaps are highlighted by oval-shaped drawings) was detected between CO and toluene. Applying the PCA method, we found that PCA1 describes approximately 94%, while PCA2 only 3.6% of data variation. These results demonstrate that PCA2 could be neglected in comparison with PCA1. Therefore, by analyzing the data projection on PCA1 to evaluate the behavior of the sensors, a high classification in clearly separated clusters is obtained for all gases via PCA. However, PCA and LDA results should be evaluated by machine learning algorithms for high accuracy. The generated feature set by PCA and LDA was provided to the classification algorithms as input vectors, Then, the training/testing process of SVM, KNN, DT, RF, and GPC classifiers was executed by using the 10-fold and 2-fold cross-validation approach. Table 2 shows the classification rate percentage of the sensor array after applying different classification algorithms.

**Table 2:** Gas classification rate.

*K*- fold cross-validation	*K* = 10		*K* = 2	
Classifier	PCA (%)	LDA (%)	PCA (%)	LDA (%)

KNN	89	69	68	55
SVM	73	77	57	61
RF	97	78	71	57
DT	96	72	65	52
GPC	99	74	85	58

**Figure 12:**
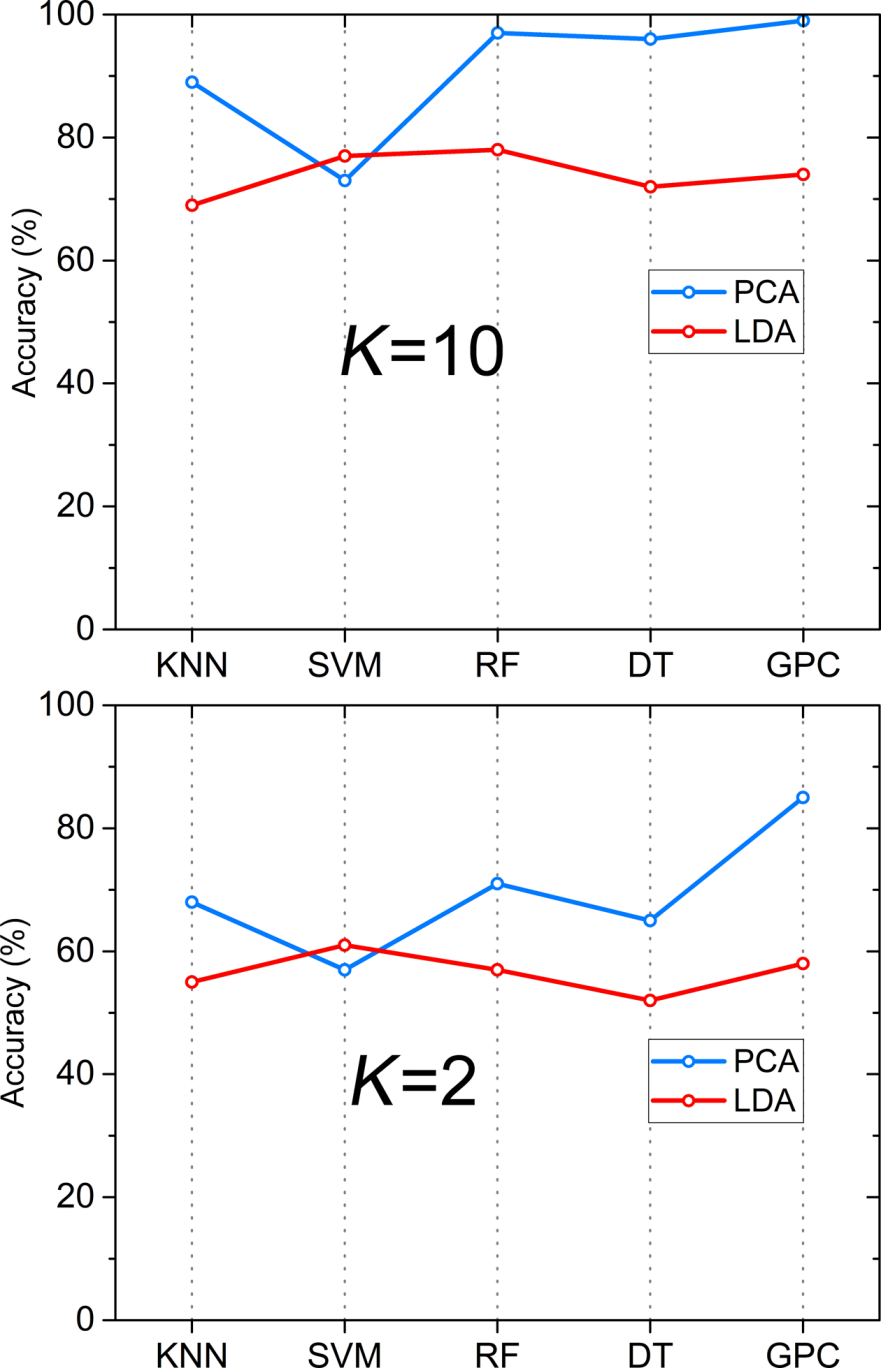
Total classification accuracy using the five classifiers SVM, KNN, DT, RF, and GPC with *K*-fold cross-validation (*K* = 2, *K* = 10).

For visualization, Figure 12 illustrates the classification accuracy of the test samples based on the presented five classifiers. The result shows that, by applying 10-fold cross-validation method, 10–90% of training and test samples were analyzed. KNN, RF, DT, and GPC classifiers yielded a higher classification rate when the PCA method was used as input vector. A different behavior was detected for SVM, where a higher classification rate was achieved by the LDA method as input vector for SVM. The highest rate with 99% classification of the target gases was achieved by using PCA as input vector to train the GPC classifier. Results show that using a smaller amount of training samples, such as the 2-fold cross-validation method in which 50% of training and test samples were analyzed, could strongly decrease the classification rate. In the case of the RF classifier, classification decreased by 30%. The same result of a 30% decrease was obtained for KNN. However, GPC still shows a very high classification rate (85%) even with 50% less training samples, which proves the high capacity and powerful discrimination of using the PCA–GPC combination for E-nose and gas sensor applications.

The usage of a powerful classification system based on statistical analysis and machine learning algorithms is a prime need for sensing applications in different fields, such as gas detection and monitoring [22–23], food industry [24], and agriculture [25]. However, the potential of such electronic nose systems varies according to the implemented classification system. Only between two and four different statistical analysis and classification algorithms were used in the cited literature [22–25]. In this work, we developed a robust system with seven different mathematical algorithms and we examined the interaction between these algorithms. The seven algorithms were studied in deep using different combinations and the highest classification rate was obtained by PCA/GPC.

## Conclusion

We presented the fabrication and characterization of seven different composite sensors based on polyaniline. These sensors were used in gas sensing arrays. The sensitivity and selectivity of the gas sensing system was successfully tested on a set of six different gases. The improvement of the selectivity was analyzed by implementation of a complex statistical classification system. The strength of statistical analysis and classification algorithms was determined and was based on several factors such as data field application, parameters, behavior of the system, and the convenience of data behavior and the data correlation regarding the mathematical algorithms and calculations. For different data patterns the algorithms show different strength and reliability. For our system it was possible to perform a suitable preprocessing and feature selection of the dataset by PCA for powerful predictions with uncertainty determined by GPC, which summarizes the distribution of random variables and define the covariance function of the data, a crucial ingredient for our system predictions. The highest classification rate of about 99% was obtained for our classification system using PCA and GPC.

## Experimental

### Sensor array fabrication

The sensor array with four interdigitated electrode systems was manufactured as a flexible printed circuit board (DuPont Pyralux AP8535 with 75 µm thickness, double-sided, copper-clad laminate in an all-polyimide composite of polyimide film bonded to copper foil). It contains the heating elements and the temperature sensors for the temperature controlling of individual sensing layers. The heating element can also be used for the desorption of measured gases or heating slightly above the ambient temperature in order to reduce temperature fluctuation. The 18 µm thick Cu interdigitated electrodes were covered with a 12 µm thick gold layer to improve the corrosion resistance. The width and spacing of interdigitated electrodes are 100 µm. The heating element (surface-mounted device SMD0402 resistor with an electric resistance of 50 Ω) and a platinum temperature sensor (SMD0603 Pt1000) are placed on the bottom side of each sensor by soldering with Sn_0.63_Pb_0.37_. The sensor size array is 16.2 mm (width) × 16.2 mm (length) with the sensing layer size of 1.7 mm × 1.7 mm. A pin header connector is used for connection of the sensor array.

**Figure 13:**
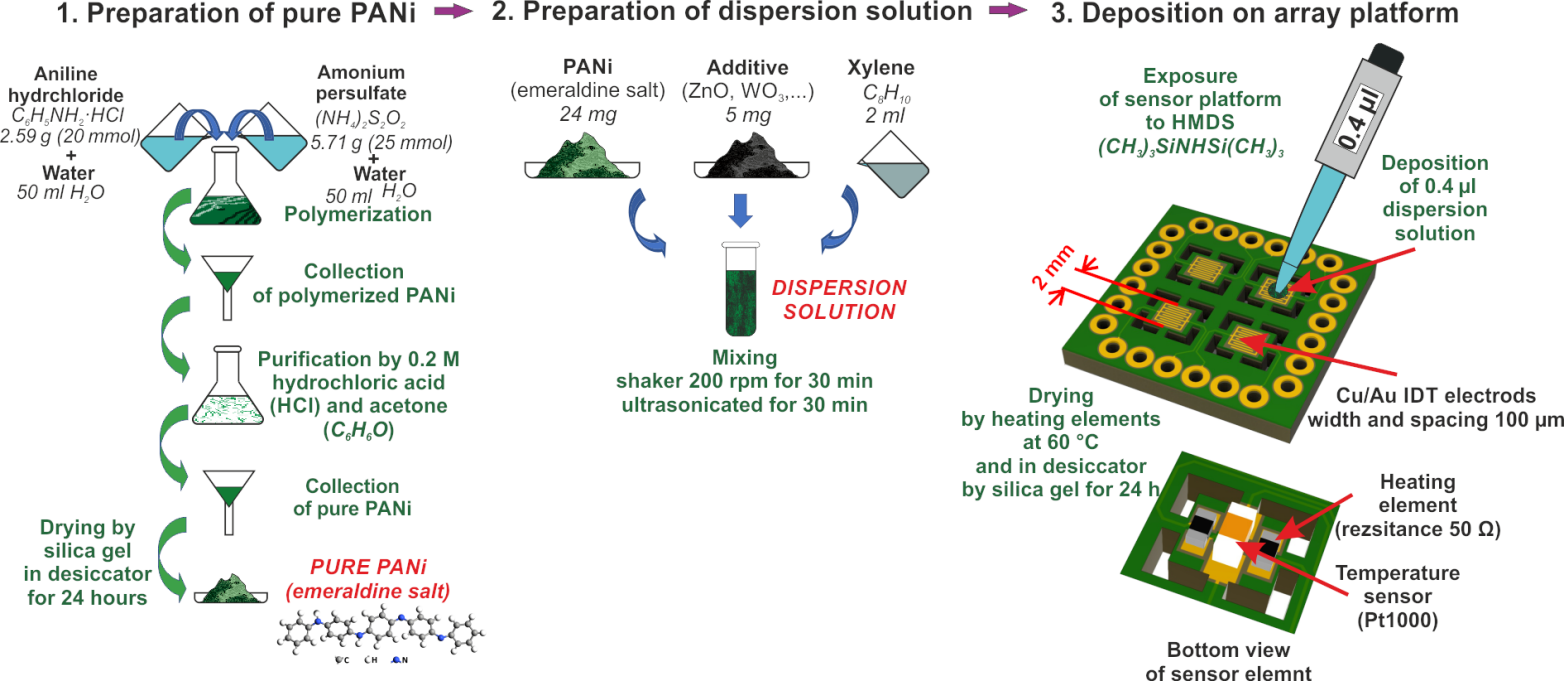
Sensor array fabrication.

Sensor arrays with PANI/nanocomposite (PANI/ZnO, PANI/WO_3_ (nanopowder), PANI/WO_3_ (nanowires), PANI/In_2_O_3_, PANI/C_60_, PANI/NCD, and PANI/BaTiO_3_) sensing layers were fabricated similarly as reported in [10]. The whole fabrication process of the sensor array is described in Figure 13.

First, the protonated emeraldine salt form of PANI was prepared by oxidation of 0.2 M aniline hydrochloride with 0.25 M ammonium persulfate at room temperature (25 °C). The precipitate obtained after the polymerization was filtered and purified by 0.2 M hydrochloric acid and acetone. Subsequently, pure polyaniline was dried over silica gel in a desiccator for 24 h.

Next, the dispersion solutions were prepared by mixing 24 mg PANI and 5 mg additives (zinc oxide, two forms of tungsten oxide, indium oxide, fullerene, NCD, and barium titanate) in 2 mL xylene. Table 3 shows properties of the used additives. The prepared solutions were mixed in a shaker for 30 min and subsequently ultrasonicated for 30 min. Finally, the obtained dispersion solutions were deposited by a micropipette on the interdigitated electrode arrays. After that, the deposited sensor layers were dried using the integrated heating elements at 60 °C for 2 h and whole sensor array was subsequently dried in a desiccator over silica gel for 24 h. Before the deposition, the sensor array platforms were cleaned in acetone and isopropyl alcohol for 15 min and then exposed to hexamethyldisilazane (HMDS) for 2 h to improve the adhesion of sensing layers.

**Table 3:** Properties of the used additives.

Additive	Properties

zinc oxide (ZnO)	nanopowder, particle size < 50 nm, surface area > 10.8 m^2^·g^−1^
tungsten oxide (WO_3_)	nanopowder, particle size < 100 nm
tungsten oxide (WO_3_)	nanowires, diameter 50 nm, length 10 µm
indium oxide (In_2_O_3_)	nanopowder, particle size < 100 nm, 99.9% trace metals basis
fullerene (C_60_)	sublimed form
nanocrystalline diamond (NCD)	nanopowder, particle size < 5 nm
barium(IV) titanate (BaTiO_3_)	nanopowder (cubic crystalline phase), particle size < 100 nm, ≥99% trace metals basis

**Figure 14:**
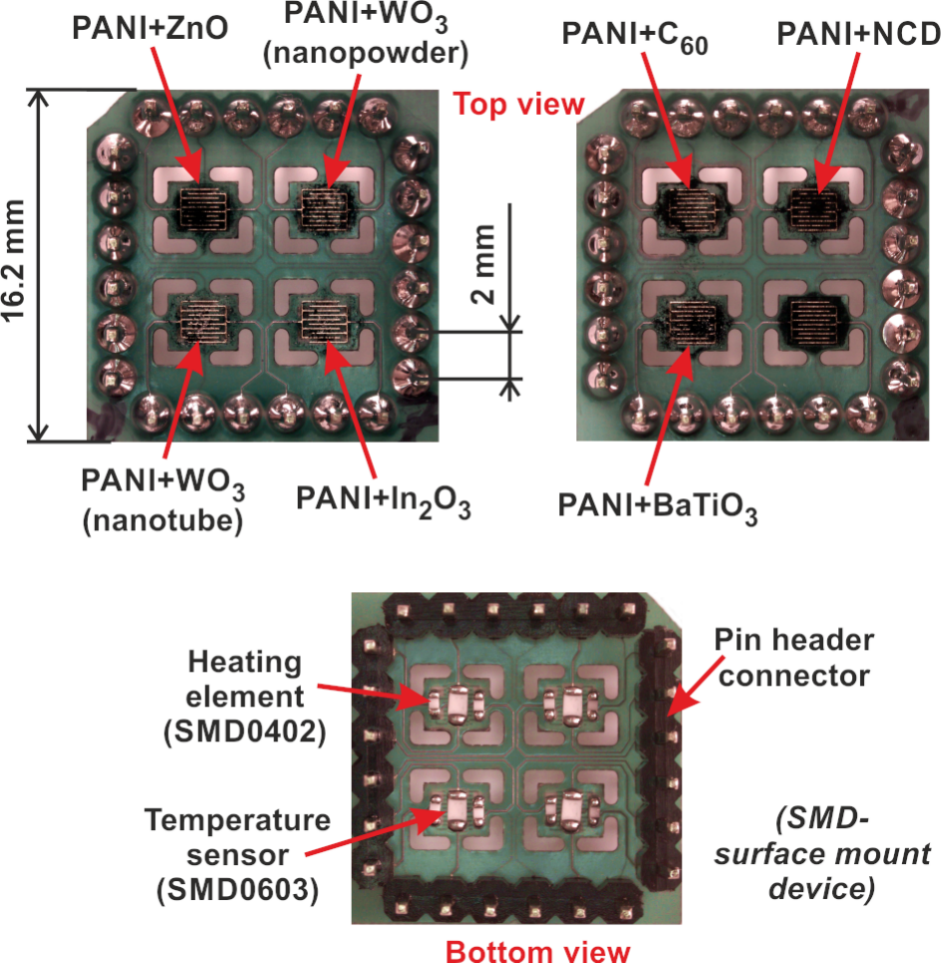
Sensor array with nanocomposite sensing layers with dimensions and bottom view with heating elements and temperature sensors.

Figure 14 demonstrates the top view of sensor array with nanocomposite sensing layers and the bottom view with heating elements and temperature sensors [26].

### Statistical classification system

**Figure 15:**
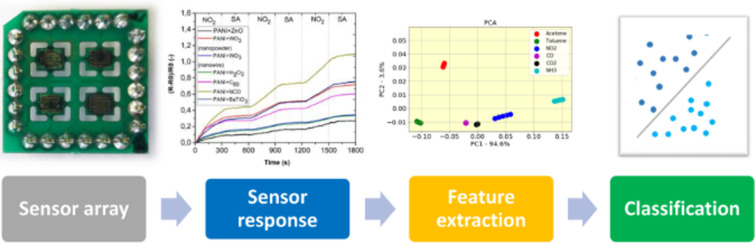
Smart sensing system block diagram.

The classification capability for the gas sensor arrays was analyzed and studied to achieve the highest classification rate by constructing a classification system according to the block diagram in Figure 15. PCA and LDA were implemented and applied in order to extract the data measured from seven sensing layers, namely PANI/ZnO, PANI/WO_3_ (nanopowder), PANI/WO_3_ (nanowires), PANI/In_2_O_3_, PANI/C_60_, PANI/NCD, and PANI/BaTiO_3_, and the result was used to train/test five different classification algorithms. In this work, KNN, SVM, RF, DT, and GPC were used to achieve the highest classification rate.

This paragraph and the following paragraph about PCA and LDA follow closely the corresponding content in [27]. PCA is a linear transformation that preserves as much data variance as possible. PCA chooses a matrix **T** that minimizes the mean squared distance between original data and those reconstructed from reduced data. It has been shown that


[2] $$T_{\text{PCA}}=U\Lambda^{-1/2},$$

where **U** and **Λ** are the eigenvector matrix and the diagonal eigenvalues matrix of the data covariance matrix, respectively. PCA has been extensively used for gas sensor applications [28–30].

LDA provides a linear projection of the data with (*c* – 1) dimensions, by considering the scatter of data within each class and across classes. Projection directions are those that maximize the inter-class separation of the projected data. The LDA transformation matrix is given by


[3]

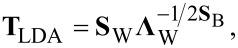



where **S**_W_ and **Λ**_W_ are, respectively, the eigenvectors matrix and the diagonal eigenvalues matrix of the within-class scatter W. **S**_B_ is the eigenvectors matrix of the between-class scatter B. LDA was previously used for gas detection applications [31–32].

KNN is a supervised algorithm that is used for classification and regression, KNN is based on the minimum distance of the unknown sample to the training samples, by selecting the specified number of points (*k*) that are closest to the unknown sample [33]. Then the unknown sample can be identified by the majority votes.

SVM is an algorithm capable of performing classification and regression. The SVM algorithm enhances the classification function by finding the hyperplane in an *N*-dimensional space that has the widest margin between training data and class boundaries. Many possible hyperplanes could be chosen, but SVM aims to find a plane with the maximum margin, that is, the maximum distance between data points of different classes in feature space. A linear decision boundary is constructed in form of a hyperplane:


[4] $$\omega^{T}x+b=0.$$

A support vector machine takes the input vectors and outputs a hyperplane that separates different classes. Hyperplanes are decision boundaries that help to classify different classes [34–35]. Samples with an unknown class will be classified by the decision boundary, samples falling on either side of the hyperplane can be attributed to different classes.

DT is a learning algorithm. It is a tree-structured classifier where the classification model is performed by a series of test questions and conditions with finite depth based on the features of input data in a tree structure. After the decision tree has been built, sorting of an unknown sample is simple. It begins with the top node of the tree and goes to the leaf. It undergoes distribution channels of the nodes and it follows to the next node, for which a new condition is subjected to the sample till it reaches the leaf node. The unknown sample is labeled and sorted, and a new and unknown sample is classified [36–37].

RF is an ensemble method for classification and regression tasks that uses multiple models of several DT to achieve a better output classification. The concept of the algorithm to build the structure is to select random samples from the dataset and create a decision tree from each sample and to get prediction results from each decision tree created [38]. The RF consists of a large number of individual decision trees. Each tree votes for a class prediction in the random forest, and the prediction with the most votes becomes the prediction of random forest.

GPC is a supervised learning method and a generalization of the Gaussian distribution of probability, which can be used for advanced non-parametric machine learning algorithms for classification problems [39]. GPC puts a Gaussian prior over a latent function, which is then squashed by a logistic function to obtain the probabilistic classification. In GPC, the posterior of the latent function is not Gaussian [40]. In GPC, for a given dataset, the training data *x*_1_,..., *x*_n_ are chosen with the corresponding class labels *y* = (*y*_1_,..., *y*_n_), to predict the class of a test sample:


[5] $$p\left(y_{i}=j|x_{i}\right)=\Phi \left(f_{i}\left(x_{i}\right)\right)\;i=1,...,n\;,\;j=1,...,k,\left(1\right),$$

where Φ denotes the standard normal cumulative distribution function, that is,


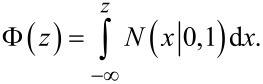


Gaussian process classification is not a parametric model. Instead, training is carried out to get hyperparameters that are needed for the covariance function and posterior. The target of GPC is to obtain the distribution of a latent variable corresponding to test sample x*:


[6]

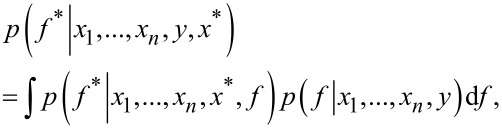



where *p*(*f*|*x*_1_, …, *x*_n_, *y*) is the posterior over the latent variables, and this distribution subsequently will be used to predict the probabilistic prediction:


[7]

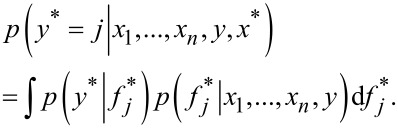



## References

[1] Hosseini S H, Khalkhali R A, Noor P (2010). Monatsh Chem.

[2] Akbar S, Dutta P, Lee C (2006). Int J Appl Ceram Technol.

[3] Korotcenkov G, Han S D, Cho B K, Brinzari V (2009). Crit Rev Solid State Mater Sci.

[4] Korotcenkov G, Cho B K (2013). Sens Actuators, B.

[5] Mao Y, Park T J, Wong S S (2005). Chem Commun.

[6] Zee F, Judy J W (2001). Sens Actuators, B.

[7] Liu S F, Moh L C H, Swager T M (2015). Chem Mater.

[8] Pugh D C, Newton E J, Naik A J T, Hailes S M V, Parkin I P (2014). J Mater Chem A.

[9] Zhang D, Liu J, Jiang C, Liu A, Xia B (2017). Sens Actuators, B.

[10] Kroutil J, Laposa A, Voves J, Davydova M, Nahlik J, Kulha P, Husak M (2018). IEEE Sens J.

[11] Zhang L, Liu Y, Deng P (2017). IEEE Trans Instrum Meas.

[12] Zhang L, Zhang D, Yin X, Liu Y (2016). IEEE Sens J.

[13] Zhang L, Tian F, Nie H, Dang L, Li G, Ye Q, Kadri C (2012). Sens Actuators, B.

[14] Kiselev I, Sommer M, Kaur Mann J, Sysoev V V (2010). IEEE Sens J.

[15] Ahluwalia A, De Rossi D (2001). Encyclopedia of Materials: Science and Technology.

[16] Salvatierra R V, Moura L G, Oliveira M M, Pimenta M A, Zarbin A J G (2012). J Raman Spectrosc.

[17] Trchová M, Morávková Z, Bláha M, Stejskal J (2014). Electrochim Acta.

[18] Nie Q, Pang Z, Lu H, Cai Y, Wei Q (2016). Beilstein J Nanotechnol.

[19] Kulkarni S B, Navale Y H, Navale S T, Stadler F J, Ramgir N S, Patil V B (2019). Sens Actuators, B.

[20] Chani M T S, Karimov K S, Khalid F A, Moiz S A (2013). Solid State Sci.

[21] Anisimov Y A, Evitts R W, Cree D E, Wilson L D (2021). Polymers (Basel, Switz).

[22] Robin Y, Goodarzi P, Baur T, Schultealbert C, Schutze A, Schneider T (2021). Machine Learning based calibration time reduction for Gas Sensors in Temperature Cycled Operation. 2021 IEEE International Instrumentation and Measurement Technology Conference.

[23] Brahim-Belhouari S, Bermak A (2005). Pattern Recognit Lett.

[24] Tan J, Balasubramanian B, Sukha D, Ramkissoon S, Umaharan P (2019). J Food Process Eng.

[25] Amkor A, Maaider K, El Barbri N (2021). Sens Actuators, A.

[26] Kroutil J (2019). Gas sensor array with nanocompositefilms.

[27] Bermak A, Belhouari S B, Shi M, Martinez D (2006). Pattern recognition techniques for odor discrimination in gas sensor array.

[28] Kermit M, Tomic O (2003). IEEE Sens J.

[29] McEntegart C M, Penrose W R, Strathmann S, Stetter J R (2000). Sens Actuators, B.

[30] Penza M, Cassano G, Tortorella F, Zaccaria G (2001). Sens Actuators, B.

[31] Aishima T (1991). J Agric Food Chem.

[32] Brahim-Belhouari S, Bermak A, Wei G, Chan P C H (2003). A comparative study of density models for gas identification using microelectronic gas sensor. Proceedings of the 3rd IEEE International Symposium on Signal Processing and Information Technology.

[33] Petersson H (2008). Multivariate Exploration and Processing of Sensor Data-applications with multidimensional sensor systems.

[34] Boser B E, Guyon I M, Vapnik V N (1992). A training algorithm for optimal margin classifiers. Proceedings of the Fifth Annual ACM Workshop on Computational Learning Theory.

[35] Cortes C, Vapnik V (1995). Mach Learn.

[36] Cho J, Li X, Gu Z, Kurup P U (2012). IEEE Sens J.

[37] Li Q, Bermak A (2011). J Low Power Electron Appl.

[38] Shi T, Horvath S (2006). J Comput Graph Stat.

[39] Yuan F, Xia X, Shi J, Li H, Li G (2017). IEEE Access.

[40] Rasmussen C E, Williams C K I (2006). Gaussian Processes for Machine Learning.

